# Diagnostic accuracy of touch imprint cytology for head and neck malignancies: a useful intra-operative tool in resource limited countries

**DOI:** 10.1186/s12907-017-0063-y

**Published:** 2017-11-25

**Authors:** Hania Naveed, Mariam Abid, Atif Ali Hashmi, Muhammad Muzammamil Edhi, Ahmareen Khalid Sheikh, Ghazala Mudassir, Amir Khan

**Affiliations:** 1 Shifa Medical College, Islamabad, Pakistan; 2 Liaquat National Hospital and Medical College, Karachi, Pakistan; 3 Brown University, Providence, RI USA; 4 Pakistan Institute of Medical Sciences, Islamabad, Pakistan; 5 Kandahar University, Kandahar, Afghanistan

**Keywords:** Touch imprint cytology, Frozen section, Head and neck malignancies

## Abstract

**Background:**

Intraoperative consultation is an important tool for the evaluation of the upper aerodigestive tract (UAT) malignancies. Although frozen section analysis is a preferred method of intra-operative consultation, however in resource limited countries like Pakistan, this facility is not available in most institutes; therefore, we aimed to evaluate the diagnostic accuracy of touch imprint cytology for UAT malignancies using histopathology of the same tissue as gold standard.

**Methods:**

The study involved 70 cases of UAT lesions operated during the study period. Intraoperatively, after obtaining the fresh biopsy specimen and prior to placing them in fixative, each specimen was imprinted on 4-6 glass slides, fixed immediately in 95% alcohol and stained with Hematoxylin and Eosin stain. After completion of the cytological procedure, the surgical biopsy specimen was processed. The slides of both touch Imprint cytology and histopathology were examined by two consultant histopathologists.

**Results:**

The result of touch imprint cytology showed that touch imprint cytology was diagnostic in 68 cases (97.1%), 55 (78.6%) being malignant, 2 cases (2.9%) were suspicious for malignancy, 11 cases (15.7%) were negative for malignancy while 2 cases (2.9%) were false negative. Amongst the 70 cases, 55 cases (78.6%) were malignant showing squamous cell carcinoma in 49 cases (70%), adenoid cystic carcinoma in 2 cases (2.9%), non-Hodgkin lymphoma 2 cases (2.9%), Mucoepidermoid carcinoma 1 case (1.4%), spindle cell sarcoma in 1 case (1.4%). Two cases (2.9%) were suspicious of malignancy showing atypical squamoid cells on touch imprint cytology, while 13 cases (18.6%) were negative for malignancy, which also included 2 false negative cases. The overall diagnostic accuracy of touch imprint cytology came out to be 96.7% with a sensitivity and specificity of 96 and 100%, respectively while PPV and NPV of touch imprint cytology was found to be 100 and 84%, respectively.

**Conclusion:**

Our experience in this study has demonstrated that touch imprint cytology provides reliable specific diagnoses and can be used as an adjunct to histopathology, particularly in developing countries, where the facility of frozen section is often not available, since a rapid preliminary diagnosis may help in the surgical management planning.

## Background

Of all human cancers, upper aerodigestive tract (UAT) malignancies are the most distressing since it is the site of most complex functional anatomy in the human body. Its importance is due to the major functional responsibilities of this region which includes breathing, taste, swallowing, voice, endocrine and cosmetic etc. Cancers that occur in this area have a major impact on these important human functions leading to difficulty in respiratory-swallowing coordination [1, 2].

Upper aerodigestive tract malignancies constitute 4-5% of all malignancies. It is the sixth most common cancer worldwide and constitutes a significant global problem with half of a million new cases diagnosed every year resulting in an average mortality rate of 7.3 and 3.2 per 100,000 males and females respectively [3]. In South East Asia, it is the second most common cancer. Pakistan falls into a high-risk head and neck cancer geographical zone with a prevalence of 22% [4–6]. Data-base of Karachi Cancer Registry have shown oral cavity as the most commonly affected site. Oral cavity carcinomas is showing a rising incidence with the mucosa cheek as the most common sub-site, followed by tongue, palate, gum, lip and floor of mouth [7].

The exact cause of cancer in this area of body is not well understood, but it is usually related to environmental and certain preventable risk factors which are self-inflicted. These include tobacco, alcohol, diet, radiation, pollution, drugs, viral infections, and other unknown factors. Amongst these, tobacco (smoked or smokeless in the form of pan and gutka chewing) is the most important, especially in our country. The prevalence of tobacco smoking in Pakistan is 36% for males and 9% for females [5, 8, 9].

In Pakistan, tobacco use is not limited to cigarette smoking. Other common forms of tobacco include water-pipe tobacco, chewing tobacco and snuff. Smokeless tobacco, whether it’s chewing tobacco or snuff, is not a safe alternative of tobacco smoking and is responsible for a higher percentage of cases of oropharyngeal carcinoma in Pakistan [10].

Intraoperative consultation remains an invaluable tool in the initial evaluation of the surgically resected specimens. It not only includes frozen section but also gross evaluation of the specimen, examination of cytology preparations taken on the specimen (e.g. touch imprints), and aliquoting of the specimen for special studies (e.g. molecular pathology techniques, flow cytometry) [11].

Intraoperative diagnosis includes frozen section and touch imprint cytology both of which provide rapid intra-operative pathologic consultation.

Frozen section procedure involves a rapid microscopic analysis of a specimen by thin slicing of tissue cut from a fresh specimen [12, 13]. Frozen section has several limitations often making the interpretation difficult. The most important limitations that interfere with the results are the technical problems which include freezing artifacts, poor quality sections, bloated cell morphology and poorly stained sections [14].

Although it is generally believed that the conventional frozen section is the best technique for intraoperative consultation, intraoperative cytology also has emerged as an accurate, simple, cheap and rapid diagnostic tool [15].

Different studies carried out in different regions of the world have proved its diagnostic accuracy. In one study imprint cytology was correlated with histological diagnosis of the corresponding biopsy from 174 patients with laryngeal and pharyngeal tumors. The imprint cytology showed a diagnostic accuracy, sensitivity, specificity, positive predictive value and negative predictive values of 97, 96, 100, 100 and 92% respectively [16].

There was another study conducted in which correlation between the biopsy specimens and touch imprint preparations in patients with 30 head and neck mass lesions were examined. The concordance between touch imprint and paraffin sections was 90%. The sensitivity and specificity of touch imprint cytology in detecting malignancy were 88 and 92%, respectively [17].

This technique has also proven its accuracy in diagnosing surgical specimens belonging to prostate, breast cancer margins and sentinel lymph nodes; however a study of this type in UAT has been lacking in Pakistan [18, 19]. Therefore we evaluated the importance of touch imprint cytology in the intraoperative consultation of UAT malignancies and this may help in avoiding a pre-operative invasive procedure. Furthermore this study is of special value is countries like Pakistan where cryostat facility is not available at many centers.

## Methods

The study was conducted in the Department of Pathology, in collaboration with the Department of Otolaryngology, PIMS, Islamabad, from 1st July 2015 to 30th march 2016 for a period of 9 months. Consecutive (non-probability) sampling was used and the sample size was calculated by using WHO sample size calculator taking:

Sensitivity = 96% [10], Specificity = 100% [10], Expected prevalence = 22% [3].

Desired Precision = 10%, Confidence level = 95%.

Sample size = 69 patients.

Inclusion criteria:

1. Patients of all ages and both genders, presenting with mass lesions or ulceration in upper aerodigestive tract as clinically or radiologically detected.
2. Patients with any duration of illness are included.
3. Patients with any co-morbid diseases (diabetes, hypertension etc. as mentioned in the history) will be eligible to be selected.

Exclusion criteria:

1. Obvious inflammatory lesions of the Upper Aerodigestive Tract as diagnosed on biopsy.

### Data collection procedure

Approval from hospital ethics committee was taken prior to the start of study. Patients were selected by consecutive sampling. After the informed consent, patients were included in the study based on lesions of upper aerodigestive tract. Intraoperatively, after obtaining the fresh biopsy specimen and prior to placing them in fixative, each specimen was imprinted on 4-6 glass slides, fixed immediately in 95% alcohol and stained with Hematoxylin and Eosin stain. The Cytology results were evaluated as

i. Malignant (Squamous cell carcinoma: isolated cells or clusters of malignant cells showing keratinization. The cells have distinct cell borders, vesicular nuclei and prominent nucleoli. Adenocarcinoma: cells are usually arranged in cohesive groups of various sizes in the form of loose clusters or acini with central lumina. The individual cells may show eccentric nuclei, mostly with prominent nucleoli and evidence of mucin production in the form of cytoplasmic vacuolation.)
ii. Suspicious for malignancy (suggestive of malignancy but uncertain due to limited number of cells or degree of atypia)
iii. Negative for malignancy (no evidence of malignancy like high N/C ratio, pleomorphism, hyperchromasia, coarse chromatin, irregular nuclear outlines).
iv. Non-diagnostic (scant cellularity, air drying or distortion artifact, obscuring blood).

After completion of the cytological procedure, the surgical biopsy specimen was immediately fixed in 10% buffered formalin. Following gross examination, the tissue was paraffin embedded. This was followed by cutting, slide preparation & staining with Hematoxylin and Eosin (H&E) stain. The Histopathology results were evaluated as:

i. Malignant (squamous cell carcinoma: sheets or nests of atypical squamoid cells having intercellular bridges and/or keratinization. Adenocarcinoma shows infiltration by malignant cells forming glandular pattern with large mucus secreting cells)
ii. Negative for malignancy (no evidence of malignancy like high N/C ratio, pleomorphism, hyperchromasia, coarse chromatin, irregular nuclear outlines seen in the biopsy material)
iii. Non-diagnostic (biopsy material inconclusive due to cautery effect or necrosis or fibrosis)

The slides of both touch imprint cytology and histopathology were examined under light microscope according to the set criteria and recorded by two consultant histopathologists and the results were recorded in a proforma.

### Data analysis

Data was analyzed using SPSS Version 10. Descriptive statistics were calculated for both qualitative and quantitative variables. Mean and standard deviation (SD) were calculated for numerical variables like age. Frequency and percentage were presented for categorical variables i.e.; gender (M: F), cytological findings and histopathological findings.

2 × 2 tables was used to determine sensitivity, specificity, positive predictive value, negative predictive value and diagnostic accuracy.

## Results

This study includes seventy cases which were collected in a 9 months period from 1st July 2015 to 30th march 2016. Random cases were selected which presented in the ENT OPD with different complains depending on the site involved. Patients with nasal tumors most commonly presented with a painless growth, sinusitis, etc. Patients with oral cavity lesions usually have non-healing ulcers, increasing growth, and difficulty in chewing etc. Laryngeal lesions usually showed hoarseness of voice, stridor, and difficulty in breathing. The patients were referred to the operation theatre for biopsy of the lesion, informed consent was taken from all the patients and the procedure of biopsy was preformed. Touch imprint slides were prepared from the fresh tissue in operation theatre.

**Fig. 1:**
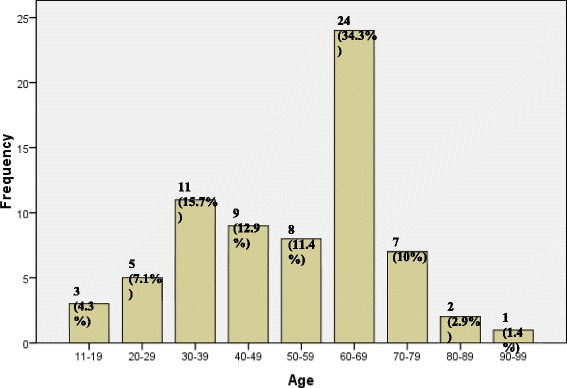
Age distribution of patients (*n* = 70)

Age distribution of the patients showed the patients range from 16 years to 93 years with the mean age being 52.87 +/− 18.26. Figure 1 demonstrates the age distribution. Majority of the patients fall in the range of 60-69 years (24 cases), followed by 30-39 years (11 cases). Of the total 70 cases, 47 cases (67.1%) were male while 23 cases (32.9%) were female. The male to female ratio is 2: 1.

**Fig. 2:**
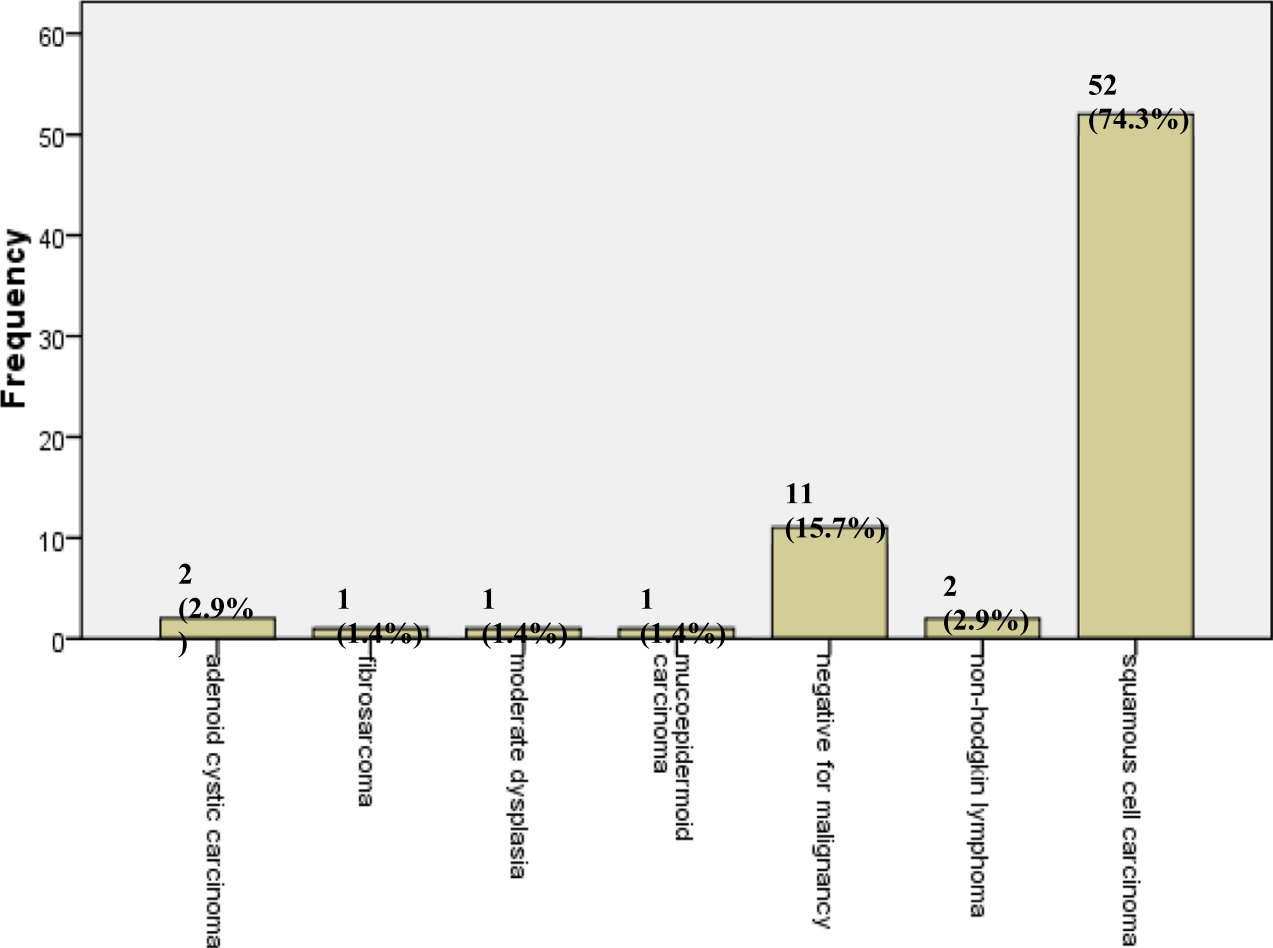
Frequency of specific Histopathologic Diagnosis (n = 70)

Of the total seventy cases, 59 cases (84.3%) were malignant, while 11 cases (15.7%) were benign. The histologic breakdown of these cases, as diagnosed on histopathology showed 52 cases (72.9%) out of 59 malignant were squamous cell carcinoma of different grades. The remaining cases include malignant salivary gland tumors comprising of 3 cases (4.3%) which included 2 cases (2.9%) of adenoid cystic carcinoma and 1 case (1.4%) of Mucoepidermoid carcinoma, non-Hodgkin lymphoma 2 cases (2.9%), fibrosarcoma 1 case (1.4%) (Table 1). One case (1.4%) also included in the malignant category was of moderate dysplasia as diagnosed on biopsy. Frequency breakdown of these cases as diagnosed on histopathology is shown in Fig. 2.

**Table 1:** Frequency of specific Touch Imprint Cytology diagnosis (*n* = 70)

Diagnosis	Frequency	Percentage
Squamous cell carcinoma	49	72.8%
Adenoid cystic carcinoma	2	2.9%
Mucoepidermoid carcinoma	1	1.4%
Non-Hodgkin lymphoma	2	2.9%
Spindle cell sarcoma	1	1.4%
Suspicious for malignancy	2	2.9%

**Fig. 3:**
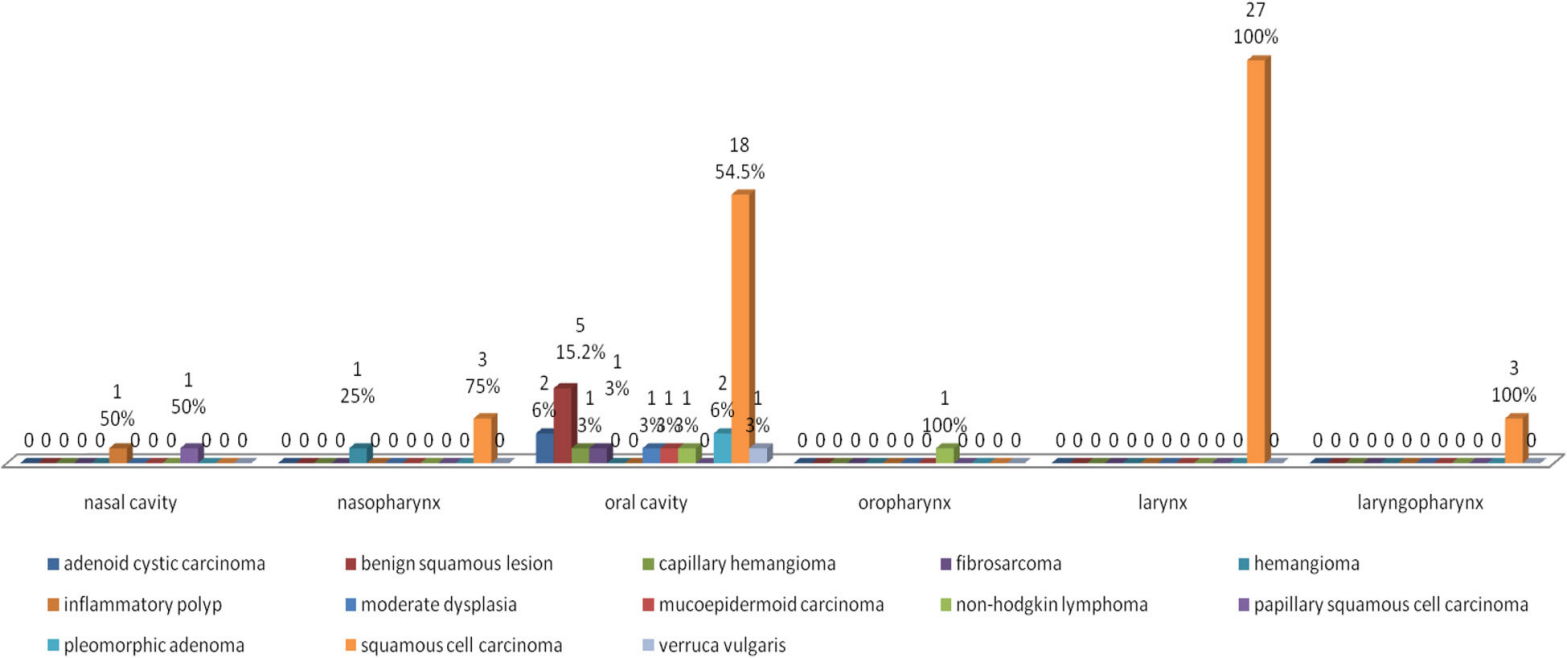
Site distribution of various types of pathologies in Upper Aerodigestive tract (*n* = 70)

The site breakdown of malignant tumors show oral cavity as the most common site of involvement, in 33 cases (47.1%), followed by larynx in 27 cases (38.6%). The nasal cavity was involved in 2 cases (2.9%), nasopharynx in 4 cases (5.7%), oropharynx in 1 case (1.4%) and laryngopharynx in 3 cases (4.3%). In the oral cavity, oral mucosa was the most commonly involved site, followed by tongue, hard palate, soft palate, tonsil, alveolar ridge and lower lip. In the larynx supraglottis is the most common site, followed by glottis, infraglottis, true vocal cord, pyriform fossa, false vocal cord and epiglottis. The site breakdowns of the cases are shown in Fig. 3.

**Fig. 4:**
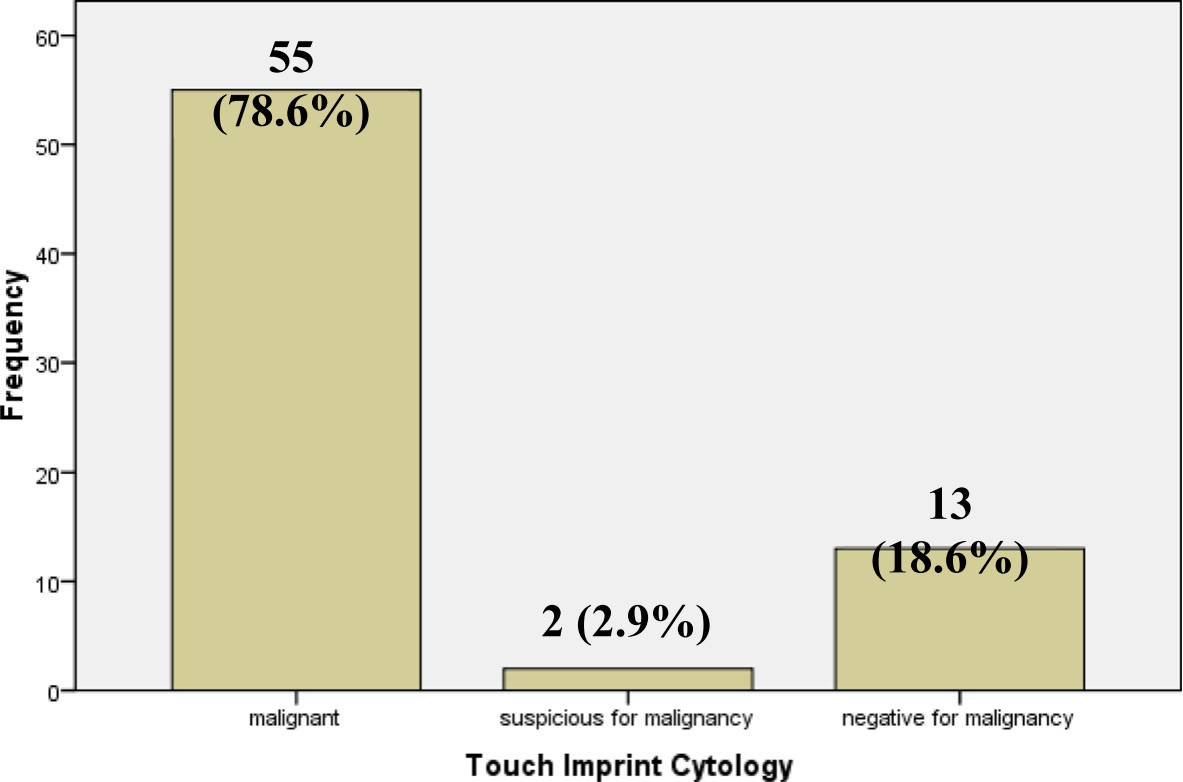
Frequency of Touch Imprint Cytology (*n* = 70)

The result of touch imprint cytology showed that touch imprint cytology was diagnostic in 68 cases (97.1%), 55 (78.6%) being malignant, 2 cases (2.9%) were suspicious for malignancy, 11 cases (15.7%) were negative for malignancy while 2 cases (2.9%) were false negative. Among the 70 cases, 55 cases (78.6%) were malignant showing squamous cell carcinoma in 49 cases (70%), adenoid cystic carcinoma in 2 cases (2.9%), non-Hodgkin lymphoma 2 cases (2.9%), Mucoepidermoid carcinoma 1 case (1.4%), spindle cell sarcoma in 1 case (1.4%). Two cases (2.9%) were suspicious of malignancy showing atypical squamoid cells on touch imprint cytology, while 13 cases (18.6%) were negative for malignancy, which also included 2 false negative cases. These statistics are shown in Fig. 4.

A strong correlation is found between the diagnosis made by touch imprint cytology and the final histopathological diagnosis as shown in Table 2. Of the 70 cases of head and neck tumors diagnosed on histopathology, complete correlation was seen in 68 cases (97.1%) cases and there was a lack of correlation in only two cases.

**Table 2:** Degree of Correlation

Degree of correlation	Frequency (%)
Complete correlation	55 (93%)
Correlation with category only	2 (3.3%)
No correlation	2 (3.3%)

The overall diagnostic accuracy, sensitivity, specificity, positive predictive value and negative predictive value of touch imprint cytology were determined for all malignant head and neck tumors. Separate statistics were calculated for squamous cell carcinoma as this was the predominant histologic type.

The overall diagnostic accuracy of Touch imprint cytology in diagnosing malignant head and neck lesions came out to be 96.7%, as shown in Table 3. The sensitivity and specificity of touch imprint cytology came out to be 96 and 100%, respectively. Whereas the PPV and NPV of touch imprint cytology was found to be 100 and 84%, respectively. 2 × 2 table for malignant aerodigestive tract lesions are shown in Table 4.

**Table 3:** Diagnostic Accuracy of Touch imprint cytology in diagnosing Malignant Upper Aerodigestive Tract lesions (*n* = 70)

Touch Imprint Cytology in Upper Aerodigestive Tract lesions	
Sensitivity	96%
Specificity	100%
Positive predictive value	100%
Negative predictive value	84%
Diagnostic accuracy	96.7%

**Table 4:** 2 × 2 table showing cases with diagnosis of Malignant Upper Aerodigestive Tract Lesions (*n* = 70)

	Histopathological Diagnosis (Malignant Upper Aerodigestive Tract Lesions)	
Touch imprint cytology	True positive (a) 57	False positive (b) 0
	False negative (c) 2	True negative (d) 11

The sensitivity and specificity of touch imprint cytology in diagnosing squamous cell carcinoma came out to be 96.2 and 100% respectively, as shown in Table 5. The PPV and NPV was 100 and 84.6%, respectively with the diagnostic accuracy being 96.8%. 2 × 2 table for squamous cell carcinoma are shown in Table 6.

The sensitivity and specificity of touch imprint cytology in diagnosing malignant salivary gland neoplasm and non-hodgkins lymphoma came out to be 100 and 100% respectively.

**Table 5:** Diagnostic Accuracy of Touch imprint cytology in diagnosing Squamous cell carcinoma (*n* = 52)

Touch Imprint Cytology in Squamous cell carcinoma	
Sensitivity	96.2%
Specificity	100%
Positive predictive value	100%
Negative predictive value	84.6%
Diagnostic accuracy	96.8%

**Table 6:** 2 × 2 table showing cases with a diagnosis of Squamous cell carcinoma (*n* = 52)

	Histopathological diagnosis (Squamous cell carcinoma)	
Touch imprint cytology diagnosis (squamous cell carcinoma)	True positive (a) 50	False positive (b) 0
	False negative (c) 0	True negative (d) 2

## Discussion

Touch imprint cytology is a well known rapid histopathological method of intraoperative analysis of biopsy specimens along with frozen section. Different studies have analyzed the importance of both procedures; however frozen sections have shown interpretational difficulties in the form of freezing artifacts, cost effectiveness, and expertise in operating the cryostat machine etc. Touch imprint cytology on the other hand is a very simple, cheap and easy to perform procedure requiring pathologist’s expertise in the cytology interpretation [20, 21].

In this study a total of seventy cases were collected over a period of 9 months. Most of the patients were referred from the E.N.T. department to operation theatre for the biopsy procedure of patient with complaints of increasing growth lesion, a non-healing ulcer, difficulty in swallowing, hoarseness of voice and stridor etc. The aim of this study was to evaluate the diagnostic accuracy of touch imprint cytology of upper aerodigestive tract malignancies in all age groups. We found that touch imprint cytology is highly sensitive and specific intraoperative procedure [22].

Most of the patients in our study were in the age range of 60-69 years comprising of 24 patients, followed by a comparatively younger age group of 30-39 years comprising of 11 cases. There were 5 cases in the range of 20-29 years, while 51 cases were above 40 years. These results are comparable to the study done by Mehrota R, who showed a head and neck cancer predominance in adults, although majority of their patients were in the range of 50-59 yrs. [23].

Our study showed a male predominance comprising of 47 males (67.1%) and 23 (32.9%) females with a male to female ratio of 2:1. The results of our study are comparable to study done by Adeyami BF et al. who analyzed head and neck cancer in a Nigerian tertiary healthcare centre. They found a male predominance in the head and neck cancers with a male to female ratio of 1.8:1. Similar findings were reported by Alverenge Lde M et al.; they also found a male predominance in carcinomas of head and neck region [24, 25].

The patients in our study usually presented with lesions in the oral cavity comprising of 33 cases (47.1%), and the second most common site is larynx comprising of 27 cases (38.6%). In the oral cavity, the oral mucosa was the most commonly involved sub-type, comprising of 16 cases and the patients usually presented with non-healing ulcer at this site. After oral mucosa, tongue is the second commonly involved site in eight patients with ulceration as the most common presentation. These results are comparable to the study conducted by Mirbod SM et al. [26].

The sensitivity and specificity of touch imprint cytology for malignant upper aerodigestive tract lesion in our study was calculated as 96% and 100% respectively. Hussein et al. conducted a similar study of touch imprint cytological preparations and head and neck lesions and they found sensitivity and specificity in detecting malignancy was 88% and 92% respectively. Comparison of intraoperative cytology and frozen section in nose and paranasal tumors were also evaluated by Nigam J et al. and they found a sensitivity, specificity and positive predictive value of 100% [10, 20].

**Fig. 5:**
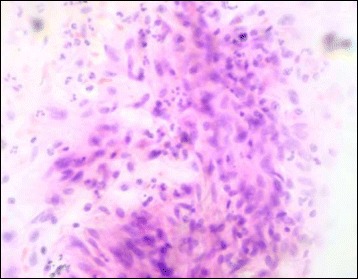
Touch imprint cytology of well differentiated squamous cell carcinoma showing cluster of neoplastic cells have vesicular nuclei, slightly pleomorphic nuclei, prominent nucleoli and abundant cytoplasm (H&E ×400)

**Fig. 6:**
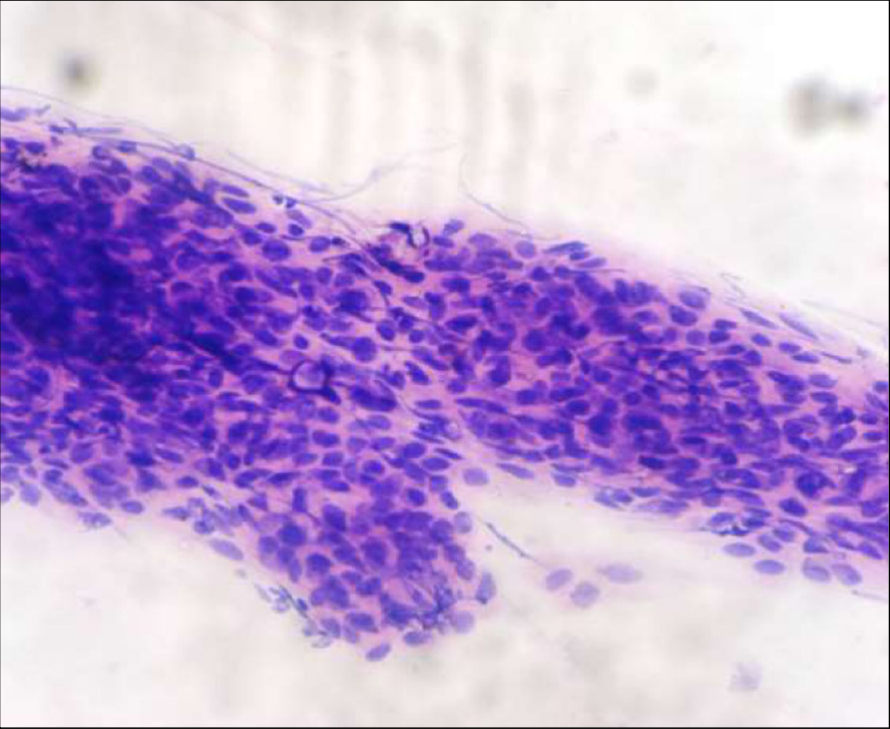
Touch imprint cytology of poorly differentiated squamous cell carcinoma showing sheets of cells having vesicular, pleomorphic nuclei and high nuclear to cytoplasmic ratio (H&E ×400)

There were a total of seventy cases, showing squamous cell carcinoma in 52 cases, which were accurately picked up by the touch imprint cytology in 50 cases. Twenty-four cases were well differentiated squamous cell carcinoma, 19 were moderately differentiated while 9 were poorly differentiated (Figs. 5 & 6).

The sensitivity and specificity of touch imprint cytology for squamous cell carcinoma in our study was 96.2 and 100% respectively. This is in accordance with the study conducted by Nieberler M et al. who evaluated the intraoperative cytology of bone resection margins in oral squamous cell carcinoma and found a high sensitivity and specificity of touch imprint cytology. Their study concluded the sensitivity and specificity of touch imprint cytology as 95.3% and 96% respectively. The positive predictive value, negative predictive value and diagnostic accuracy of their study came out to be 93.8, 96.9 and 95.7% respectively [27].

**Fig. 7:**
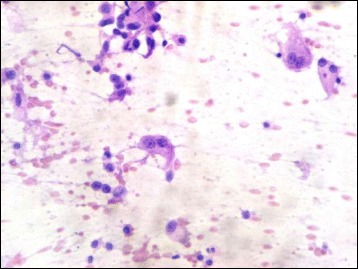
Touch imprint cytology of Mucoepidermoid carcinoma showing intermediate cells and a mucin filled cell (H&E ×400)

**Fig. 8:**
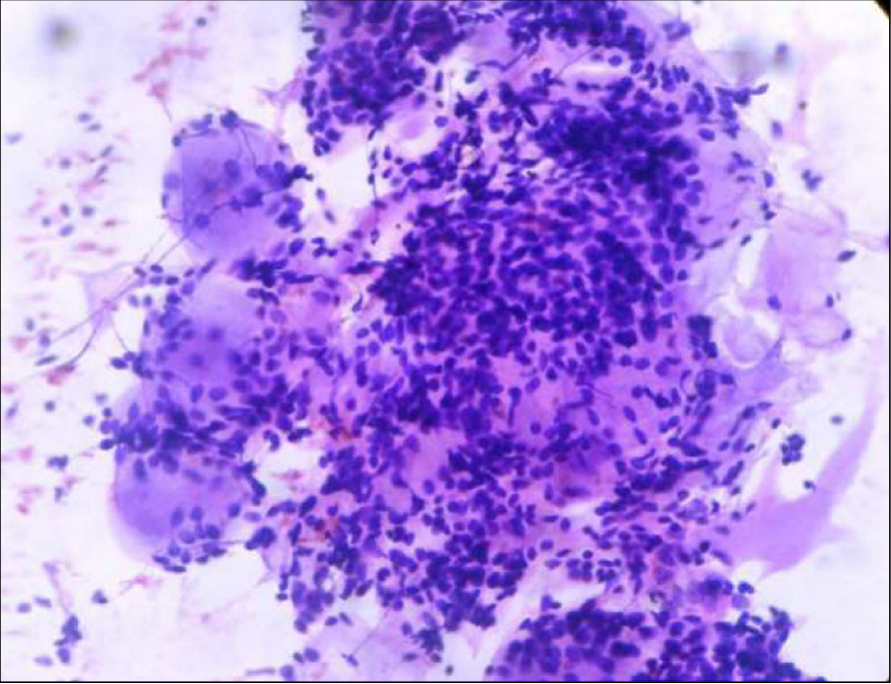
Touch imprint cytology of adenoid cystic carcinoma showing cribriform arrangement of basaloid cells having hyperchromatic, angulated nuclei. Basophilic secretions characteristic of adenoid cystic carcinoma are also seen (H&E ×400)

Three cases of malignant salivary gland neoplasm were also encountered. They included one intermediate grade Mucoepidermoid carcinoma and two cases of adenoid cystic carcinoma. Touch imprint cytology accurately picked up both cases with a sensitivity, specificity, positive predictive value, negative predictive value and diagnostic accuracy of 100%. On touch imprint cytology of Mucoepidermoid carcinoma there were cells with typical squamoid appearance mimicking a squamous cell carcinoma, however there were admixed large cells with abundant, mucin filled cytoplasm, thereby confirming a diagnosis of Mucoepidermoid carcinoma (Fig. 7). Adenoid cystic carcinoma on the other hand showed cells arranged in a cribriform architecture on touch imprint cytology. The neoplastic cells have hyperchromatic, angulated nuclei and scant cytoplasm (Fig. 8). All three cases aroused from the hard palate which is most common site for minor salivary gland neoplasm. Jansisyanont P et al. in their experience of intraoral minor salivary gland tumors also found palate as the most frequently involved site by minor salivary gland neoplasms [28].

There were two cases of non-Hodgkin lymphoma, both involved the oral cavity. One case involved the tonsil while the other involved the oral cavity. Both cases on touch imprint cytology showed sheet of large atypical lymphoid cells having with increased nuclear size, vhirregular chromatin clumping, prominent nucleoli and scant cytoplasm. Atypical mitotic figures were also seen. On permanent sections both cases were diagnosed as non-Hodgkin lymphoma.

There was one case of spindle cell sarcoma, diagnosed as fibrosarcoma after the application of immunohistochemistry on histologic sections. The sarcoma involved the oral mucosa showed sheets of atypical spindle shaped cells having hyperchromatic, pleomorphic nuclei with irregular nuclear contours and chromatin on cytology smears. Frequent atypical mitoses were also seen. Resection specimen of the same case showed spindle cell sarcoma in a herring bone pattern, which on immunohistochemistry was negative for all specific lineage markers like smooth muscle actin, desmin, myogenin, pancytokeratin etc.

Two cases were given suspicious of malignancy, due to atypical nuclear features but the cellularity was too low to make a definitive diagnosis. One of the case had atypical cells admixed with benign squamoid cells misleading the diagnosis. On permanent sections well differentiated squamous cell carcinoma was diagnosed. The other case showed mature squamoid cells with mild nuclear changes in the form of hyperchromasia, nuclear membrane abnormalities, but the changes were not significant to diagnose it as a malignant smear. Permanent sections of the same case showed only moderate dysplasia of the lining epithelium. No invasive carcinoma was identified. Since touch imprint cytology shows only cytological features, architectural changes in the form of basement membrane invasion cannot be commented upon. This is the drawback of touch imprint cytology that invasion cannot be identified; however touch imprint cytology can detect dysplastic cytological changes.

There were two cases which showed benign morphology on touch imprint cytology, while the permanent sections showed malignant squamous cell carcinoma. One case on touch imprint cytology showed benign squamoid cells which on permanent sections showed sections lined by stratified squamous epithelium with only a small focus showing dysplastic epithelial changes and invasion into the sub-epithelial tissue. This small focus was not sampled in touch imprint cytology giving a false negative diagnosis. The problem can be solved by making multiple smears from different biopsy sides so that all the cut surfaces are touched on the slides, minimizing the false negative results. The other case showed very hypocellular smears with mostly inflammatory cells. On permanent sections well differentiated squamous cell carcinoma was present along with ulceration of the epithelium.

## Conclusion

Intraoperative consultation is an important tool for the evaluation of the lesions, specifically the nature of the lesion, adequacy of the biopsy, margin status etc. This study demonstrates that touch imprint cytology is an important tool for the evaluation of malignant upper aerodigestive tract lesions.

Although architectural features are subtle and focal in touch imprint cytology smears but the specific cytological features can help in avoiding diagnostic pitfalls. The smears should always be carefully interpreted by a trained cytopathologist to avoid misdiagnosis. Multiple smears should be made from the specimen after carefully dabbing the specimen surface to avoid blood obscuring artifacts and low cellularity etc. The clinical findings and other diagnostic modalities, like radiological imaging should always be known before interpretation of the lesion.

Our experience in this study has demonstrated that touch imprint cytology provides important diagnostic information that can play a significant role in patients management, especially by differentiating between neoplastic and reactive lesions. Moreover, in a majority of cases, it can provide reliable specific diagnoses and can be used as an adjunct to histopathology, particularly in developing countries like ours, where the facility of frozen sections is often not available, since a rapid preliminary diagnosis may help in surgical management planning.

## References

[1] Uppaluri R, Dunn GP, Lewis JS (2008). Focus on TILs: prognostic significance of tumor infiltrating lymphocytes in head and neck cancers. Cancer Immun.

[2] Givens DJ, Karnell LH, Gupta AK, Clamon GH, Pagedar NA, Chang KE (2009). Adverse events associated with concurrent chemoradiation therapy in patients with head and cancer. Arch Otolaryngol Head Neck Surg.

[3] Masood N, Kayani MA (2011). Mutational analysis of xenobiotic metabolizing genes (CYP1A1 and GSTP1) in sporadic head and neck cancer patients. Genet Mol Biol.

[4] Chaudhry S, Khan AA, Mirza KM, Iqbal HA, Masood Y, Khan NR (2008). Estimating the burden of head and neck cancers in the public health sector of Pakistan. Asian Pac J Cancer Prev.

[5] Goon PK, Stanley MA, Ebmeyer J, Steinstrasser L, Upile T, Jerjes W (2009). HPV and head and neck cancer a descriptive update. Head Neck Oncol.

[6] Slootweg PJ, Richardson M, Gnepp DR (2009). Squamous cell carcinoma of the upper aerodigestive system. Diagnostic surgical pathology of head and neck.

[7] Bhurgri Y, Bhurgri A, Usman A, Pervez S, Kayani N, Bashir I (2006). Epidemiological review of head and neck cancers in Karachi. Asian Pac J Cancer Prev.

[8] Lee YC, Marron M, Benhamou S, Bouchardy C, Ahrens W, Pohlabeln H (2009). Active and involuntary tobacco smoking and upper aerodigestive tract cancer risks in a multicenter case-control study. Cancer Epidemiol Biomark Prev.

[9] hmed R, Rashid R, McDonald PW, Ahmed SW. Prevalence of cigarette smoking among young adults in Pakistan. JPMA. 2008;58(11):597–601. 19024129

[10] Bile KM, Shaikh JA, Afridi HU, Khan Y. Smokeless tobacco use in Pakistan and its association with oropharyngeal cancer. East Mediterr Health J. 2010;16 Suppl:S24-30. 21495585

[11] Wenig BM (2008). Intraoperative consultation (IOC) in mucosal lesions of the upper Aerodigestive tract. Head Neck Pathol.

[12] Hashmi AA, Naz S, Edhi MM, Faridi N, Hussain SD, Mumtaz S, Khan M (2016). Accuracy of intraoperative frozen section for the evaluation of ovarian neoplasms: an institutional experience. World J Surg Oncol.

[13] Hashmi AA, Faridi N, Khurshid A, Naqvi H, Malik B, Malik FR, Fida Z, Mujtuba S (2013). Accuracy of frozen section analysis of sentinel lymph nodes for the detection of Asian breast cancer micrometastasis - experience from Pakistan. Asian Pac J Cancer Prev.

[14] Thomson AM, Wallace WA (2007). Fixation artifacts in an intra-operative frozen section: a potential cause of misinterpretation. J Cardiothorac Surg.

[15] Khalid A, Haque AU (2004). Touch imprint cytology versus frozen section as intraoperative consultation diagnosis. Int J Pathol.

[16] Loncar B, Pajtler M, Milicić-Juhas V, Kotromanović Z, Staklenac B, Pauzar B (2007). Imprint cytology in laryngeal and pharyngeal tumors. Cytopathology.

[17] Hussein MR, Rashad UM, Hassanein KA (2005). Touch imprint cytological preparations and the diagnosis of head and neck mass lesions. Ann Oncol.

[18] Aytac B, Atalay FO, Vuruskan H, Filiz G (2012). Touch imprint cytology of prostate core needle biopsy specimens: a useful method for immediate reporting of prostate cancer. J Cytol.

[19] Esbona K, Li Z, Wilke LG (2012). Intraoperative imprint cytology and frozen section pathology for margin assessment in breast conservation surgery: a systematic review. Ann Surg Oncol.

[20] Jaafar H (2006). Intra-operative frozen section consultation: concepts, applications and limitations. Malays J Med Sci.

[21] Nigam J, Misra V, Dhingra V, Jain S, Varma K, Singh A (2013). Comparative study of intra-operative cytology, frozen sections, and histology of tumor and tumor-like lesions of nose and paranasal sinuses. J Cytol.

[22] Mehanna H, Paleri V, West CM, Nutting C (2010). Head and neck cancer--part 1: epidemiology, presentation, and prevention. BMJ.

[23] Mehrotra R, Singh M, Gupta RK, Singh M, Kapoor AK (2005). Trends of prevalence and pathological spectrum of head and neck cancers in North India. Indian J Cancer.

[24] Adeyemi BF, Adekunle LV, Kolude BM, Akang EE, Lawoyin JO (2008). Head and neck cancer--a clinicopathological study in a tertiary care center. J Natl Med Assoc.

[25] Alvarenga Lde M, Ruiz MT, Pavarino-Bertelli EC, Ruback MJ, Maniglia JV, Goloni-Bertollo M (2008). Epidemiologic evaluation of head and neck patients in a university hospital of Northwestern São Paulo state. Braz J Otorhinolaryngol.

[26] Mirbod SM, Ahing SI (2000). Tobacco-associated lesions of the oral cavity: part II. Malignant lesions. J Can Dent Assoc.

[27] Nieberler M, Häusler P, Drecoll E, Stoeckelhuber M, Deppe H, Hölzle F (2014). Evaluation of intraoperative cytological assessment of bone resection margins in patients with oral squamous cell carcinoma. Cancer Cytopathol.

[28] Jansisyanont P, Blanchaert RH, Ord RA (2002). Intraoral minor salivary gland neoplasm: a single institution experience of 80 cases. Int J Oral Maxillofac Surg.

